# Interfacial Reaction and IMC Growth of an Ultrasonically Soldered Cu/SAC305/Cu Structure during Isothermal Aging

**DOI:** 10.3390/ma11010084

**Published:** 2018-01-06

**Authors:** Yulong Li, Weifeng Long, Xiaowu Hu, Yanshu Fu

**Affiliations:** 1 Key Lab of Robot & Welding Automation of Jiangxi Province, Mechanical & Electrical Engineering School, Nanchang University, Nanchang 330031, China; lwfeng121321@163.com (W.L.); huxiaowu@ncu.edu.cn (X.H.); yshfu@ncu.edu.cn (Y.F.); 2 State Key Laboratory of Advanced Welding and Joining, Harbin Institute of Technology, Harbin 150001, China

**Keywords:** isothermal aging, intermetallic compound, ultrasonic bonding

## Abstract

In order to accelerate the growth of interfacial intermetallic compound (IMC) layers in a soldering structure, Cu/SAC305/Cu was first ultrasonically spot soldered and then subjected to isothermal aging. Relatively short vibration times, i.e., 400 ms and 800 ms, were used for the ultrasonic soldering. The isothermal aging was conducted at 150 °C for 0, 120, 240, and 360 h. The evolution of microstructure, the IMC layer growth mechanism during aging, and the shear strength of the joints after aging were systemically investigated. Results showed the following. (i) Formation of intermetallic compounds was accelerated by ultrasonic cavitation and streaming effects, the thickness of the interfacial Cu_6_Sn_5_ layer increased with aging time, and a thin Cu_3_Sn layer was identified after aging for 360 h. (ii) The growth of the interfacial IMC layer of the ultrasonically soldered Cu/SAC305/Cu joints followed a linear function of the square root of the aging time, revealing a diffusion-controlled mechanism. (iii) The tensile shear strength of the joint decreased to a small extent with increasing aging time, owing to the combined effects of IMC grain coarsening and the increase of the interfacial IMC. (iv) Finally, although the fracture surfaces and failure locations of the joint soldered with 400 ms and 800 ms vibration times show similar characteristics, they are influenced by the aging time.

## 1. Introduction

In the field of electronic packaging, solders play a crucial role in achieving electronic, thermal, and mechanical continuity of electronic components [1]. Sn-Pb solders have been the primary filler materials in electronic packaging, owing to their superior performances and low cost [2,3]. Recently, however, lead-free solder alloys have gradually replaced Sn-Pb solders, because of public concerns about the environmental and health hazards of Pb [4,5]. 

Among lead-free solders, high-Ag-content Sn-Ag-Cu solder alloys such as Sn-3 wt % Ag-0.5 wt % Cu (SAC305) is a potential substitute, owing to its good mechanical properties, good solderability, and wettability with current surface finishes [4,6]. However, SnAgCu solders possess a higher melting point compared to lead solders, necessitating a relatively long soldering time and causing a thermal expansion coefficient mismatch between the solder and the electronic components. This facilitates thermal distortion on the joint and thus limits their application in electronic packaging. The long-term reliability of SnAgCu solder joints is insufficient to meet the demands of miniaturization and multi-functionalization in electronic packaging [7,8], especially in military, geophysical logging, and space applications. To solve the above issues, numerous research efforts have been made to enhance the reliability of the solder joint by changing the joining materials or joining methods.

One of the current efforts to improve the properties of joints involves adding other particles into the lead-free solders [9]. Many researchers have found that the wettability of lead-free solders and the mechanical properties of the soldering joint can be strengthened by adding metal or rare earth particles into the solder matrix [9,10,11,12,13,14]. However, the superior properties of these composite solders are primarily dependent on the degree of dispersion of the particles in the solders, but uniform dispersion of particles in the solders is difficult because of the aggregation and small solubility in the solders of the particles [14]. Additionally, adding other particles into SnAgCu solders may induce unpredictable microstructural changes in the course of operation.

In addition to the above-mentioned methods, some studies have recently reported that a reliable solder joint with a high-temperature re-melting point can be obtained via an ultrasonic bonding process [15,16,17]. In the work of Li et al., Cu/Sn foil/Cu solder joints with homogeneous intermetallic compound obtained for ultrasonic bonding in the range of 1 s to 4 s, but some defects can be observed in the joints [15]. Xiao et al. examined the effect of ultrasonic vibration on the properties of a Cu/Zn-Al/Cu solder joint, finding that solder joints with better shear strength could be obtained through ultrasonic-assisted brazing for 4 s [17]. Although promising results have been reported, research into ultrasonic bonding is still in initial stages and needs to be more extensively studied.

In the present work, a Cu/SAC305/Cu structure was soldered using the ultrasonic spot welding method to rapidly form homogeneous IMC joints at room temperature. The research is focused on the effect of isothermal aging on microstructural evolution and mechanical properties in terms of the tensile shear strength of the joint acquired with different ultrasonic bonding times. This includes (i) analysis of the interfacial reaction and IMC formation during soldering processes, (ii) the evolution of IMCs during isothermal aging, and (iii) the effects of aging time on the mechanical properties.

## 2. Experimental Procedures

**Figure 1:**
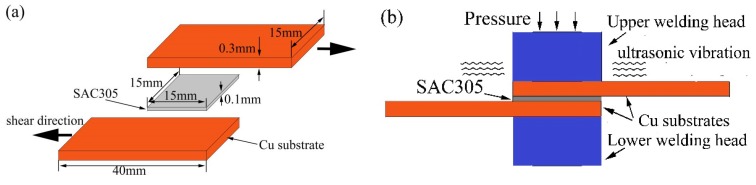
(**a**) Schematic of the Cu/SAC305/Cu sandwich structure and (**b**) the ultrasonic bonding principle.

In this study, a Cu/SAC305/Cu sandwich bonding structure was adopted. It is shown in Figure 1a. A SAC305 foil was placed between the Cu plates. The SAC305 foil had dimensions of 1.5 mm × 1.5 mm × 100 µm and the Cu plates with dimensions of 40 mm × 15 mm × 0.3 mm were used as the substrates. Prior to bonding, all the surfaces to be joined were first etched in dilute acid and then in NaOH solution to remove the oxides.

Figure 1b illustrates the principle of ultrasonic bonding with the bonding force and ultrasonic vibrations. The ultrasonic bonding parameters are an ultrasonic frequency of 20 kHz, a pressure of 0.2 MPa, and a power of 500 W, respectively. The bonding time was 400 ms and 800 ms, relatively short vibration time, to prevent the overproduction of IMCs. After ultrasonic bonding, the specimens were aged in a vacuum furnace at 150 °C for 0, 120, 240, and 360 h, respectively. The obtained ultrasonically bonded specimens mounted in epoxy were ground with 600#, 1200#, 1500#, and 2000# grade silicon carbide papers and then polished with 2.5, 1.5, and 0.5 μm diamond pastes.

The microstructure of the joints were observed with a Scanning Electron Microscope (SEM; FEI Quanta 200F, Eindhoven, Netherlands) equipped with an energy-dispersive X-ray spectrometer (EDS, IE250X Max50, Oxford Instruments, Oxford, England), which was used for imaging, analysis of microstructural features, and identification of the compositions of the reaction phases. The scanning acceleration voltage was 20 kV. The tensile shear strengths of the joints were measured by a bond tester (INSTRON 1100, Instron China Company, Shanghai, China) with a shear speed of 100 µm s^−1^, and the average strength was determined from four specimens acquired under the same conditions. The fracture morphologies of the joints after the tests were examined with the SEM using secondary electron scanning. 

The equivalent thicknesses of the IMCs were measured using the SEM image of the metallographic cross-sections and an image analysis procedure detailed as follows. (i) Firstly, a SEM image of the sample was obtained at an appropriate magnification. (ii) Secondly, the grayscale of the SEM image was enhanced using SISC IASV 8.0 image analysis software (Beijing ZhongKe KeYi computing technology Company, Beijing, China) to identify the interfaces between the different layers and their pixels. (iii) The mean value of the equivalent thickness (*L*_IMC_) of the individual layer was then calculated using the following equation:

*L*_IMC_ = (*N*_IMC_/*N*_SEM_) × *L*_SEM_ (1)

in which *L*_SEM_ is the actual height of the individual SEM image, and *N*_IMC_ and *N*_SEM_ are the number of pixels in the IMC layers and the entire SEM image, respectively.

## 3. Results and Discussion

### 3.1. Microstructure Analysis

**Figure 2:**
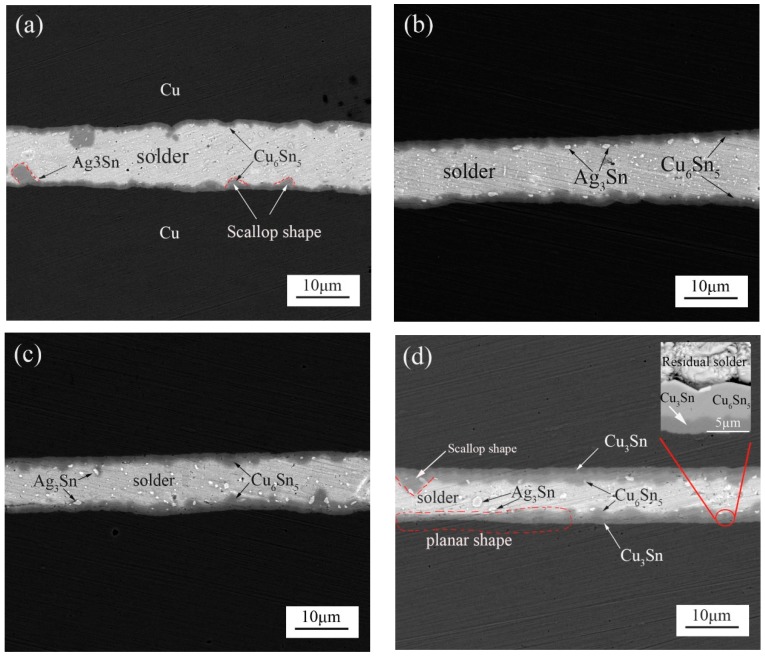
The morphologies of the cross-sectional Cu/SAC305/Cu joints formed at a 400-ms ultrasonic bonding time at different aging times of (**a**) 0 h; (**b**) 120 h; (**c**) 240 h and (**d**) 360 h. The inset in (d): the local enlargement image at the interfaces.

**Figure 3:**
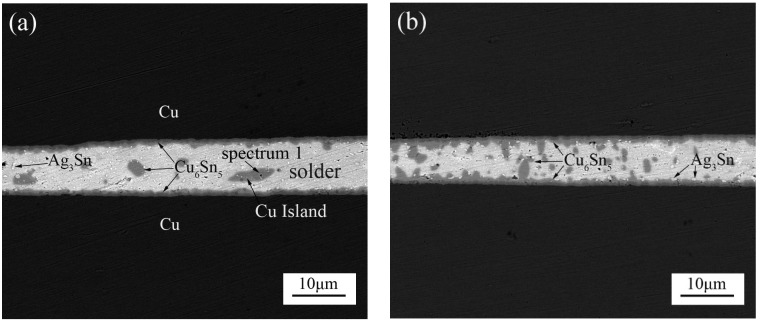

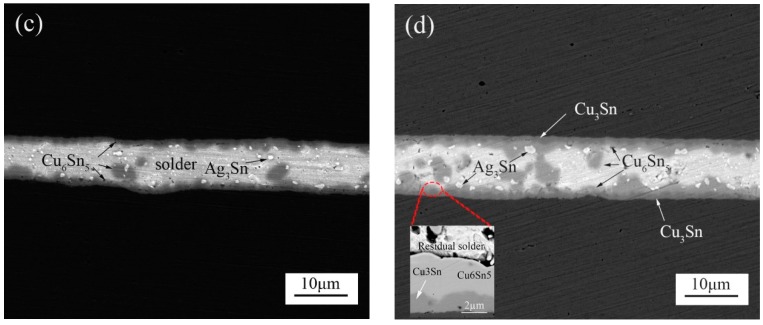
The morphologies of the cross-sectional Cu/SAC305/Cu joints formed at an 800-ms ultrasonic bonding time at different aging times of (**a**) 0 h; (**b**) 120 h; (**c**) 240 h and (**d**) 360 h. The inset in (d): the local enlargement image at the interfaces.

Figure 2a displays the SEM images of the cross-sectional Cu/SAC305/Cu soldered joints formed with a 400-ms ultrasonic bonding vibration time. The resultant joints contained residual solders and thin Cu_6_Sn_5_ layers distributed at the interfaces of the residual solders/substrates. Some light particles of Ag_3_Sn distributed randomly inside the residual solder bulk or mounted in the Cu_6_Sn_5_ layer. The thicknesses of the Cu_6_Sn_5_ layers at the top and bottom interfaces were measured to be approximately 2.36 µm and 2.38 µm, respectively. Ultrasonic bonding is a joining method using ultrasonic vibration and friction to generate a heat source, which can quickly form a high quality joint at room temperature. In our study, the SnAgCu solder is quickly melted and reacted with the Cu substrate during soldering. Ultrasonic wave can accelerate the convection between the atoms of joint during soldering. In this case, the Cu atoms in the molten solder are rapidly supersaturated, which lays the foundation for the formation and growth of IMC island. Meanwhile, the local hot spot can be generated in the interfaces and molten solder because of the ultrasonic effect [15], and then quickly cool and solidify to form a reliable joint [18,19,20,21,22]. Figure 2b–d show the interfacial microstructure of Cu/SAC305/Cu solder joints after isothermal aging at 150 °C for various durations ranging from 120 to 360 h. The thickness of the interfacial IMC layer increased after aging for 120 h and 240 h, as shown in Figure 2b,c. With the aging time increasing, morphological changes of the interfacial IMC layers is not definitive from the results of Figure 2a,d. Much scallop structure can be observed in Figure 2a. However, compared to Figure 2a, besides the scallop structure, the planar structure can be observed clearly in Figure 2d. The randomly distributed Ag_3_Sn particles were seen to grow into larger pebble-like particles from the results of Figure 2a,d. A new Cu_3_Sn phase was identified between the Cu_6_Sn_5_ IMC layer and the Cu substrate, as can be seen from the images inserted in Figure 2d and Figure 3d (the images inserted in Figure 2d and Figure 3d were obtained after corrosion). Hu et al. reported the reaction equation of Cu_6_Sn_5_ transforms into Cu_3_Sn as follows [23]:

Cu_6_Sn_5_ + 9Cu→5Cu_3_Sn (2)

Figure 3 shows the microstructure of the joints with an ultrasonic bonding time of 800 ms and isothermal aging at different holding times. The thickness of the IMC layers increased with the increase of aging time, as illustrated in Figure 3b–d. Compared with the microstructure of the joints soldered with a 400 ms ultrasonic bonding time, a number of randomly distributed small IMC islands inside the residual solders were observed, as shown in Figure 3a–d. The IMC island can be identified to be Cu_6_Sn_5_ by using EDS (as seen in Table 1). Due to the existence of IMC islands, the morphological changes of interfacial IMC layers are much more complicated than Figure 2. With the aging time increasing, the irregular shape IMC islands are gradually connected with the interfacial IMC. Ultrasonic effect promotes more Cu atoms of the substrate to go into the solder with a longer ultrasonic vibration. Meanwhile, ultrasound effects promote the homogenization of Cu atoms and the localized heat dissipation induces the formation of randomly distributed Cu_6_Sn_5_ islands within the solder joint. Additionally, the randomly distributed small Cu_6_Sn_5_ phase will also grow and aggregate to form a much larger phase during aging, as shown in Figure 3b–d. In this case, the larger IMC islands would significantly affect joint strength, which will be discussed in the following sections.

### 3.2. Growth Mechanism of the Total IMC Layer

In order to study the growth mechanism of the IMC layer at the top and bottom interfaces, a simple power-law equation was used for the analysis, as shown below:

*X* = *X*_o_ + *Kt^n^* (3)

where *X* is the average thickness of the IMC layer at time *t*, *X*_o_ is the original thickness, *K* is the growth rate constant, *n* is the time exponent, and *t* is the aging time. The Equation (3) can be converted into a logarithmic expression as:

In(*X* − *X*_o_) = *n*In*t* + In*K* (4)

The time exponent (*n*) can be obtained from the slope of curves of In(*X* − *X*_o_) against lnt. the time exponents (*n*) are approximated to 0.5 and linear correlation coefficients of these plots were greater than 0.98 after fitting. Similar results can be seen in the studies of Kim et al. and Abdelhadi et al. [24,25]. Thus, an equation can be obtained:

*X* = *X*_o_ + *Kt*^1/2^ (5)

**Figure 4:**
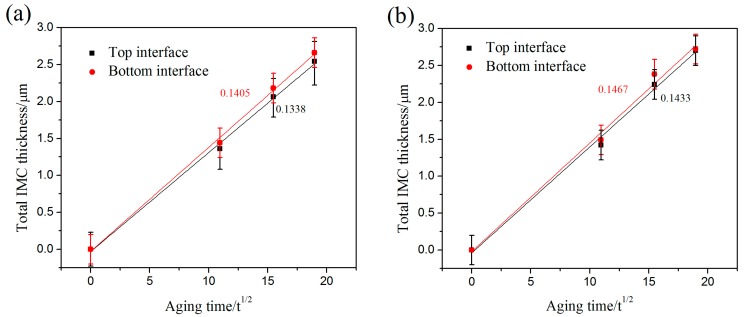
The total Cu_6_Sn_5_ and Cu_3_Sn thickness of the top and bottom interfaces with aging time at different bonding times: (**a**) 400 ms and (**b**) 800 ms.

The growth rate *K* was obtained from the linear slope of *X* − *X*_o_ versus *t*^1/2^. Figure 4 shows the growth rate of the interfacial IMC layers of the top interface and bottom interface as the aging time increased from 0 h to 360 h. When the ultrasonic bonding time was 400 ms, the growth rate of the IMC layers of the top interface and bottom interface were 3.72 × 10^−17^ m^2^/s and 3.90 × 10^−17^ m^2^/s, respectively. When the ultrasonic bonding time increased to 800 ms, the growth rate of the IMC layers of the top interface and bottom interface were 3.98 × 10^−17^ m^2^/s and 4.07 × 10^−17^ m^2^/s, respectively. Compared to the results of Ji et al., the joints with fewer defects have been obtained under small parameters [16]. Meanwhile, as seen in Figure 3d, the IMC islands also maintain a relatively stable growth rate until the IMC islands connect to the IMC layer of the upper and bottom interfaces. The joints in the aging process still maintain a good micro-morphology.

### 3.3. Effect of Aging on Shear Strength

**Figure 5:**
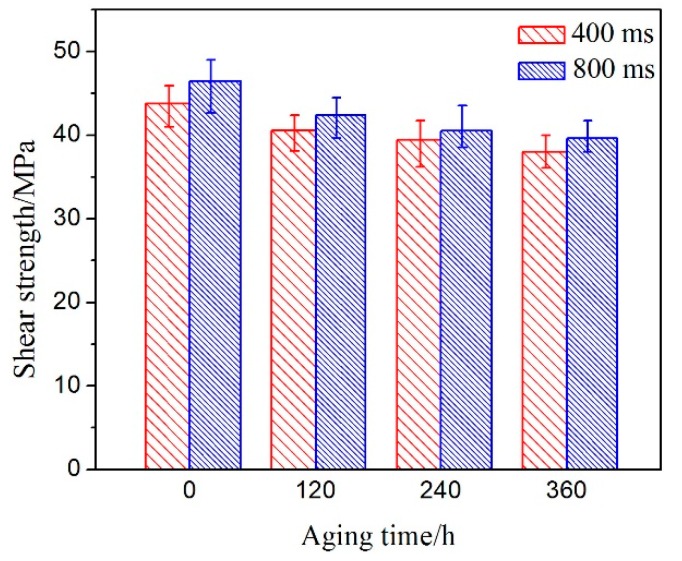
Shear strengths of joints after aging under different bonding times.

The tensile shear test is a traditional method for detecting the reliability of a solder joint in electronic packaging. Figure 5 shows the shear strengths of the soldering samples acquired at different ultrasonic vibration times and after isothermal aging at 150 °C for various durations. The shear strengths of the samples decrease as the isothermal aging time increases from 0 to 360 h. Meanwhile, the shear strength decreased noticeably at the initial stage of aging and then displayed a slight decline during further aging, and the shear strength of the solder joints with 400 ms ultrasonic bonding time was lower than that of the solder joints at 800 ms with the same aging time. In the case of a 400 ms ultrasonic bonding time, the maximum and minimum values of the shear strength were 43.80 MPa and 37.96 MPa, respectively, and 46.50 MPa and 39.60 MPa were reached with an ultrasonic bonding time of 800 ms.

A number of studies have found that the thickness of IMC layers between solder and metal substrates increases noticeably with increasing aging time, resulting in a decline in shear strength of the solder joints [4,7]. From the results of Zeng et al., the inherent brittleness and the tendency to form structural defects of a very thick IMC layer may weaken the reliability of the solder joint [26]. However, contrary conclusions were also obtained in previous investigations. Yoon et al. and Li et al. reported that the shear force has no significant relationship with IMC thickness during isothermal aging [4,27,28]. In general, the increase in average thickness of the IMC layer will influence the shear force during isothermal aging, owing to the well-known intrinsic brittleness of the Cu-Sn IMC. Additionally, the shear strength of the solder joint is reduced because of the coarsening of the solder bulk microstructure as fractures occur inside the solder. For the 400 ms ultrasonic bonding time, the decrease in shear strength with increasing aging time may be caused by the growth of Ag_3_Sn particles. For the 800 ms ultrasonic bonding time, the Cu_6_Sn_5_ island within the solder bulk significantly reduced the shear strength of the solder joint. Meanwhile, the ultrasonic shock wave with a longer vibration time can effectively contribute to the uniform dispersion of small Ag_3_Sn and Cu_6_Sn_5_ particles inside the soft solder. After soldering, the particles can strengthen the shear strength of the solder. It is reasonable to explain that the shear strength of solder joints at 800 ms is higher than that of the solder joint with the 400 ms soldering time [16,29]. 

**Figure 6:**
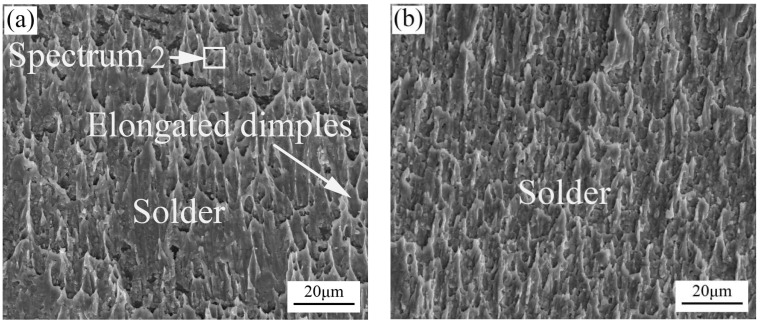

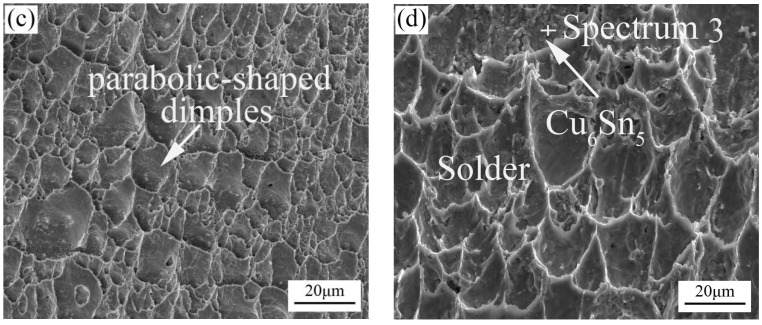
Top views of the shear fracture surfaces for the solder joints at a 400-ms ultrasonic bonding time for different aging times: (**a**) 24 h; (**b**) 120 h; (**c**) 240 h and (**d**) 360 h.

Figure 6 shows the top views of the fracture surfaces of the ultrasonically soldered joint with a 400 ms soldering time and ultrasonically soldered joints after different aging times at 150 °C. The SEM fractograph of the original ultrasonically soldered joint displayed a fractured surface with many typical elongated shear dimples, as shown in Figure 6a. The dimples along the loading direction on the shear fracture surface indicate a ductile fracture occurred under the experimental shear test condition. As shown in Table 1, the compositions of the fracture surface are 94.76 wt % Sn, 4.36 wt % Ag, and 0.88 wt % Cu. It can be inferred that the solder joint fractured inside the solder bulk. As shown in Figure 6b–d, for aging times of 120 h, 240 h, and 360 h, respectively, elongated and parabolic-shaped ductile dimples can be observed on the fracture surfaces. The joints fractured mainly inside the solder bulk, which is analogous to the fracture behavior of the initial soldered joint. Meanwhile, as shown in Figure 6d, some of the IMC particles can be observed to be due to the solder being pulled out; these were identified to be Cu_6_Sn_5_ using EDS (as seen in Table 1).

**Figure 7:**
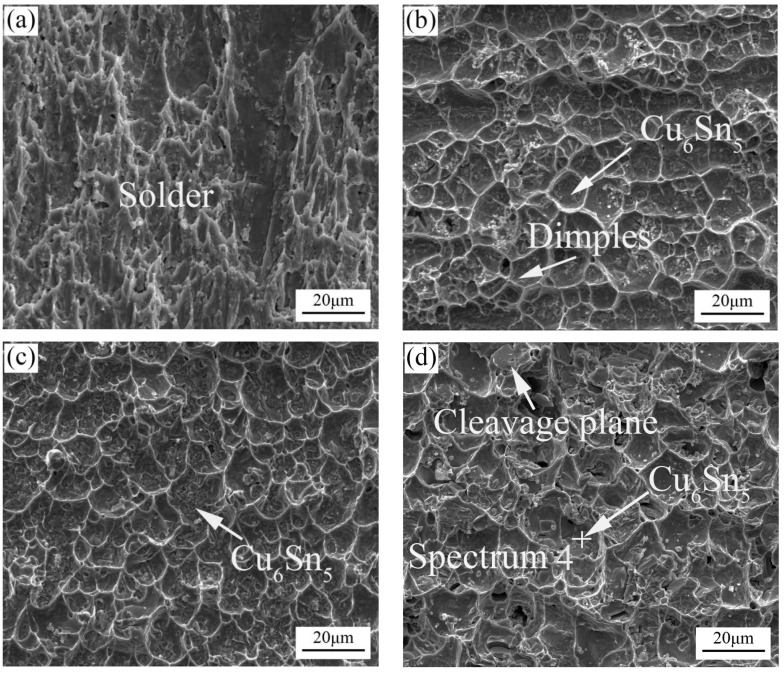
Top views of the shear fracture surfaces for the solder joints at 800-ms ultrasonic bonding times for different aging time: (**a**) 24 h; (**b**) 120 h; (**c**) 240 h and (**d**) 360 h.

**Figure 8:**
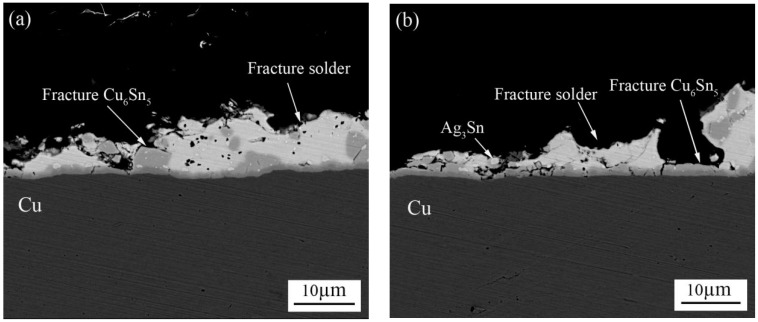
Fracture modes in solder joints formed at an 800-ms ultrasonic bonding vibration time for aging times of (**a**) 240 h and (**b**) 360 h.

Figure 7 shows SEM micrographs for the fracture surfaces of the ultrasonically soldered joints with 800-ms ultrasonic bonding times aged at 150 °C for 0 h, 120 h, 240 h, and 360 h. The typical dimples can be seen on the fracture surface after 800 ms ultrasonic bonding, as shown in Figure 7a. The dimples on the fracture surface of the solder joint display evidence of plastic deformation along the loading direction. Figure 7b,c show detailed fractographs of solder joints aged for 120 h and 240 h, respectively. Compared to the fracture surfaces of 400 ms ultrasonic soldering time, numerous bowl-shaped dimples are arranged close together at the fractured surface and the surface consists of residual solder and exposed Cu_6_Sn_5_ particles in Figure 7b–d. Different from the typical plastic fracture in 400 ms ultrasonic soldering after aging, the fracture pattern is biased towards hybrid fracture in 800 ms ultrasonic soldering after aging. However, the phenomenon of brittle fracture is more obvious with the increase of aging time, ductile dimples and cleavage plane can be observed easily in Figure 7d. Meanwhile, as seen in Figure 8a,b, the cross-sectional microstructure of the fractured solder joints aged for 240 h and 360 h are displayed, respectively. It is clear that the fracture pattern is still hybrid fracture containing brittleness and plasticity. The nature of the fracture may not change much. Therefore, the shear strength did not change much with increasing aging time.

## 4. Conclusions

After ultrasonic vibrations were applied to a Cu/SAC305/Cu bonding structure, the evolution of microstructure, the growth mechanism of the IMC layer, and the shear strength of the joint after isothermal aging at 150 °C for different times were studied, and the findings can be summarized as follows:

(1) A single Cu_6_Sn_5_ IMC layer was observed at the interfaces after the ultrasonic bonding process, and a Cu_3_Sn IMC layer was also formed at the interface between the Cu_6_Sn_5_ IMC and the Cu substrate because of the reactions between Cu_6_Sn_5_ and Cu after isothermal aging. With 800 ms of ultrasonic vibration, some small Cu_6_Sn_5_ islands were generated and aggregated into a larger island during subsequent isothermal aging as a result of supersaturated Cu in the solder bulk.
(2) During isothermal aging at 150 °C, the IMC layers of the top and bottom interfaces became thicker. The growth of the IMC was diffusion-controlled and fitted parabolic growth kinetics. For a 400-ms soldering time, the growth rates of the top and bottom interfaces were respectively 3.72 × 10^−17^ m^2^/s and 3.90 × 10^−17^ m^2^/s. When the bonding time was 800 ms, the growth rates of the top interface and bottom interface were respectively 3.98 × 10^−17^ m^2^/s and 4.07 × 10^−17^ m^2^/s.
(3) The shear strength of the solder joints with a 400-ms ultrasonic bonding time was lower than that of solder joints with an 800-ms bonding time. The critical reason for this was that the uniform dispersion of small Ag_3_Sn and Cu_6_Sn_5_ particles inside the soft solder could strengthen the solder joints. The shear strength of the solder joints exhibited a slight decline with increasing aging time because of the coarsening of IMC particles and the thickening of the IMC layer. The dimples on the fracture surfaces of the solder joints show evidence of ductile failure behavior with a 400 ms ultrasonic bonding time. After an 800 ms ultrasonic bonding time, the fracture pattern shown a composite fracture containing brittleness and plasticity and tensile shear strength tends to be stable with increasing aging time.

## Figures and Tables

**Table 1:** The EDS analysis.

Sites	Main Elements (at %)			Phase
	Ag	Sn	Cu	
1	—	43.83	56.17	Cu_6_Sn_5_
2	4.36	94.76	0.88	Sn-3%Ag-0.5%Cu
3	—	44.64	55.36	Cu_6_Sn_5_
4	—	47.25	52.75	Cu_6_Sn_5_
